# Williams syndrome: A surprising deficit in oromotor praxis in a population with proficient language production

**DOI:** 10.1016/j.neuropsychologia.2014.11.032

**Published:** 2015-01

**Authors:** Saloni Krishnan, Lina Bergström, Katherine J. Alcock, Frederic Dick, Annette Karmiloff-Smith

**Affiliations:** a Centre for Brain and Cognitive Development, Department of Psychological Sciences, Birkbeck, University of London, UK; b Institute of Cognitive Neuroscience, UCL, UK; c Department of Psychology, Lancaster University, UK

**Keywords:** Orofacial movements, Sequencing, Motor ability, Speech motor control, Williams syndrome

## Abstract

Williams Syndrome (WS) is a neurodevelopmental disorder of known genetic origin, characterized by serious delays in language onset yet relatively verbose, intelligible and fluent speech in late childhood and adulthood. How do motor abilities relate to language in this group? We investigated planning and co-ordination of the movement of the speech articulators (oromotor praxis) in 28 fluent-speaking individuals with WS, aged between 12 and 30 years. Results indicate that, despite their fluent language, oromotor praxis was impaired in WS relative to two groups of typically-developing children, matched on either vocabulary or visuospatial ability. These findings suggest that the ability to plan, co-ordinate and execute complex sensorimotor movements contribute to an explanation of the delay in expressive language early in development in this neurodevelopmental disorder. In the discussion, we turn to more general issues of how individual variation in oromotor praxis may account for differences in speech/language production abilities across developmental language disorders.

## Highlights

• Individuals with Williams Syndrome are impaired when imitating oral movements.
• Visual presentation of movements hampers performance more than aural presentation.
• Initial oromotor deficits may contribute to surprising early language delays.

## 1 Introduction

Williams Syndrome (WS) is a neurodevelopmental disorder caused by a hemizygous submicroscopic deletion of some 28 contiguous genes on chromosome 7q11±23 (Ewart et al., 1993; Donnai and Karmiloff-Smith, 2000). Although original estimates of the prevalence of WS were around 1:20,000 (Kaplan et al., 2001; Morris et al., 1988; Donnai and Karmiloff-Smith, 2000), a more recent study rates prevalence at close to 1:7,500 (Strømme et al., 2002). WS is associated with cardiac problems, distinctive facial morphology and slow physical growth. The linguistic profile of individuals with WS is typified by relatively verbose, fluent speech from late childhood onwards, a characteristic all the more striking given the fairly considerable delay in language development over infancy and toddlerhood (Singer-Harris et al., 1997; Paterson et al., 1999). However, very little is known about oromotor praxis (that is, the ability to plan and co-ordinate movements of the speech articulators) in WS, a motor ability that is particularly important for speech and language development. In the current study, we investigate oromotor praxis in a group of 12–30-year-olds with WS to establish whether oromotor ability is typical or atypical in this unusual neurodevelopmental disorder.

We first review studies that indicate that expressive language is a relative strength in the cognitive profile of WS, we then discuss why motor skills may be relevant to language development in this group, and finally, we outline the measures used in the present study.

### 1.1 Expressive language in WS

Individuals with WS present with an uneven and unusual cognitive profile. In adulthood, the language abilities of individuals with WS are usually better than their spatial cognition skills (Donnai and Karmiloff-Smith, 2000; Jarrold et al., 1998). Udwin and Yule (1990) studied conversational exchanges of 43 school-age children with WS. Eighty-four percent of these children were classified as having fluent, articulate speech. In a direct comparison of children with WS to children with specific language impairment (SLI) or Down Syndrome (DS), Laws and Bishop (2004) found that children with WS between the ages of 6–15 years outperformed the other two disorder groups on the speech sub-scale of the Childhood Communication Checklist. Other evidence for expressive language strength comes from studies of oral narrative production in WS, where the stories of individuals with Williams syndrome were more descriptive and engaging than the stories of those with DS (Reilly et al., 1990).

Within language, phonological skill generally, and phonological short-term memory more specifically, are considered to be strengths in WS (Vicari et al., 1996; Nichols et al., 2004). Relative to children with DS, children with WS performed better on tasks relying on phonological short-term memory such as digit span (Wang and Bellugi, 1994; Jarrold et al., 1999), word span (Vicari et al., 2004), or verbal repetition (Vicari et al., 2002). This was the case despite similar or poorer performance by individuals with WS on visuo-spatial memory tasks such as the Corsi block span. While reliance on phonology was originally hypothesized to be unusual in WS (Vicari et al., 1996), more recent studies suggest that phonology is a relative strength but not atypical (Majerus et al., 2003; also reviewed in Brock, 2007). Indeed, phonological abilities are comparable to those of typically developing children matched on verbal or nonverbal skill (Brock, 2005; Grant et al., 1997, Laing et al., 2005).

Despite later strengths in expressive language, during the infant and toddler years, individuals with WS present with very clear delays in language development. The onset of the first words is delayed in infants with WS and tends to occur between 18 and 24 months of age (Masataka, 2001). Parental questionnaires indicate that infants with WS have similar levels of word production and comprehension as infants with DS (Singer-Harris et al., 1997). Furthermore, they produce fewer manual gestures (such as pointing) than infants with DS (Laing et al., 2002; Singer-Harris et al., 1997). In experimental studies, infants with WS have shorter looking times to named objects relative to chronological age-matched controls and their performance resembles that of children with DS (Paterson et al., 1999). Nazzi et al. (2003) observed that although infants with WS could segment words with a strong–weak stress pattern in fluent speech, they were delayed when they had to extract words with a weak–strong stress pattern from fluent speech. Therefore, it is clear that infants with WS have early delays in lexical and phonological development. Delays in abilities relevant to language continue at later stages of development, for example, toddlers with WS are impaired in triadic joint interaction as well as comprehension and production of pointing (Laing et al., 2002). Differences in language development are observed even in the preschool years, for instance, preschoolers with WS are slower at word learning than their typically developing peers (Havy et al., 2010). Vicari et al. (2004) show that the strengths in receptive vocabulary and sentence repetition typically associated with WS only emerge by late childhood/ adolescence. It remains unclear why these initial delays arise in language development and how children with WS overcome them to become relatively proficient language producers later in development.

### 1.2 Links between language and motor abilities

In other neurodevelopmental disorders where speech and language deficits have been identified, concomitant motor difficulties are frequently observed. For example, Brookman et al. (2013) have reported poorer imitation of body postures and hand movements in SLI (also see Hill, 2001). Fine motor ability in the early years has been found to predict later speech fluency in children with autism (Gernsbacher et al., 2008; LeBarton and Iverson, 2013). Leonard and Hill (2014) have suggested that genetic disorders like Williams syndrome offer an opportunity to understand relationships between motor and language abilities through the lifespan. Yet, in contrast to the increasing literature on motor abilities in behaviourally‐defined developmental disorders like autism (Torres et al., 2013), relatively little is known about oromotor abilities in WS.

A motor ability that we refer to as ‘oromotor praxis’ is an index of an individual's ability to imitate and sequence complex oral movements. Oromotor praxis relates to language development at ages beyond the measures of motor control taken in infancy. In typically developing children of around 21 months of age, oromotor praxis is associated with scores on language production, comprehension and grammatical complexity (Alcock and Krawczyk, 2010). Further, our own research has identified links between oromotor praxis and nonword repetition, one which lasts through the school years (Krishnan et al., 2013a) and suggests that this relationship taps into the reliance of both tasks on planning and coordinating oral movements. Even in atypically developing children, oromotor praxis appears to be associated with language outcomes. For instance, a link between oromotor praxis and phonological skill is seen in specific language impairment (Stark and Blackwell, 1997) and Elliott et al. (1990) report deficits of oromotor praxis in DS. Given that speech fluency is considered a characteristic strength in this neurodevelopmental disorder (Rossi et al., 2011), it is of particular interest to establish whether oromotor praxis ability relates to verbal ability in WS. In particular, this would allow us to explore whether the emergence of relatively good oromotor skills could influence the improvement in language proficiency.

While a handful of studies indicate that infant motor milestones are delayed in WS (Lenhoff et al., 1997; Masataka, 2001; Tsai et al., 2008), very little is known about speech motor ability or oromotor praxis in children, adolescents and adults with WS. To date, one unpublished study indicates that fine motor control of the speech articulators is affected (Mervis and Velleman, 2011). However, it has not been established if oromotor ability is related to the strengths in verbal ability. As the discrepancy between verbal and visuospatial ability only appears to develop over time (Vicari et al., 2004), it is possible that strengths in oromotor ability may only be apparent at the same time or slightly earlier than strengths in verbal ability. Furthermore, strengths in oromotor praxis may only emerge over time. As children with WS have a proclivity for social interaction, their interest in conversation may lead them to imitate words and sentences more than other children with developmental disorders. It is plausible that greater experience producing speech (relative to other children with neurodevelopmental disorders) could contribute to the improvement in oromotor praxis, as children gain increased practice with sequencing and coordinating articulators to produce sounds and words in their own language. Additionally, developmental improvements in phonological proficiency might also shape and change oromotor co-ordination for speech. Currently, it is not known what levels of oromotor ability individuals with WS attain by the time verbal strengths are apparent. In addition to the previously described strengths in spoken language, speech fluency is a characteristic strength in this group and this strength is apparent by relatively early childhood (Rossi et al., 2011). Therefore, in the current study, we have focused on oromotor skills in older individuals with WS who would be likely to show the relative strengths in spoken language and speech fluency. Consequently, we expected to see concomitant strengths in oromotor skills for individuals with WS with verbal mental ages approximating those of 7–12 year olds.

### 1.3 The present study

In this study, we compare oromotor praxis in individuals with WS to both vocabulary age-matched and visuospatial age-matched controls. Given reported strengths in speech fluency in childhood in WS, we expected that oromotor praxis would be at a par with typically developing children of similar verbal ability and better than typically developing with similar visuospatial ability.

In addition to the comparison of oromotor praxis across groups, we explore whether potential group differences will be reflected across other manual, oral and verbal tasks (visuomotor imitation, oral diadochokinetic tasks and nonword repetition). Using multiple tasks that make differential demands on visuospatial, verbal, and motor skills allows us to build a profile of the strengths and weaknesses associated with memory, phonology, and motor skill in this group. We include measures of phonological and visuospatial short-term memory to ascertain whether memory demands in either domain influence oromotor praxis. A speeded oral diadochokinetic task is used to assess whether potential differences in oromotor praxis are consistent with broader oromotor ability. Diadochokinetic tasks test the ability to perform rapid repetitive muscle movements of the arm, hand or fingers and are an important aspect of neurological examination; these movements require a reversal of the pattern of reciprocal innervation of agonists and antagonists (Ziegler, 2002). Similarly, rapid repetitions of a syllable such as ‘puh’, ‘tuh’ or ‘kuh’ are used to assess the muscular system for speech (Robin et al., 1997). Nonword repetition is assessed to explore whether the demands of the oromotor praxis task are similar to those for putting together familiar phonological syllables in a novel sequence.

## 2 Methods

Prior to testing, informed consent was obtained from all participants. The study received approval from the Birkbeck Research Ethics Committee.

### 2.1 Participants

Twenty-eight children and adults with WS (CA: 12.7–28.4 years; mean: 16.5 years; 11 females) were recruited via the Williams Syndrome Foundation, UK. All participants had previously been clinically diagnosed with WS and had their diagnosis confirmed by a positive fluorescent in situ hybridisation testing (FISH) test for the Elastin deletion on one copy of chromosome 7.

We were fortunate to have already-collected, rich datasets from two cohorts of typically developing (TD) children. These TD data have been reported previously (Krishnan et al., 2013a, under review). Both these groups of TD children had also completed the sight word reading efficiency subtest of the Test of Word Reading Efficiency (TOWRE); and had standard scores above 80; indicating no overt learning/reading difficulties and confirming that they were developing typically. According to parental report, the TD children had no speech, language, hearing or academic difficulties.

Participants with WS were compared to these two previously tested cohorts of TD children, the first consisting of 39 children (CA: 7.1–12.5 years; mean: 10.1 years; 19 females) and the second of 35 children (CA: 5.4–8.6 years, mean: 7.1 years; 15 females). The age range of the first TD cohort corresponded with the verbal mental age of the WS group (assessed via the BPVS-II, Dunn et al., 1997). The ‘verbal mental age’ of the WS group ranged between 6.0 and 14.4 years (mean: 9.8 years) and was not significantly different from the chronological age of the TD children in this cohort (*p*=0.529). This TD cohort is henceforth referred to as the VMA (vocabulary age-matched) controls. The age range of the second cohort was comparable to the WS participants in terms of visuospatial mental age (assessed by the Ravens Colored Progressive Matrices, Raven et al., 1992). The participants with WS performed similarly to 5.5–10.5 year olds (mean: 7.4 years) on this visuospatial task; this was not significantly different to the ages of the TD children in the second cohort (*p*=0.173). Henceforth, this cohort is referred to as the VSMA (visuospatial age-matched) controls.

Other researchers (Jarrold and Brock, 2004) have commented on the limitations of using control groups matched on age (rather than IQ measures). As our control groups were previously-tested cohorts rather than individually matched children, we could not obtain the same normative measures. We did confirm that the children in our control groups were TD using standard scores on the TOWRE, which associates strongly with aspects of language ability such as syntax (Leech et al., 2007), phonology (Nation and Hulme, 2010) and vocabulary (Ricketts et al., 2007). Further, large sample sizes and extensive behavioural testing for the control groups also allow us to assess individual variability in TD children, which remains important in this age range (Krishnan et al., 2013b; Leech et al., 2007).

### 2.2 Procedure

Participants performed all the tasks listed below in a single session, with multiple breaks. A subset of the WS participants was also part of a longitudinal study. These participants completed attentional (Test of Everyday Attention in Children, Manly et al., 2001) and academic achievement measures (Weschler Objective Numerical Dimensions, Wechsler, 1996) in a second session (typically 2–4 weeks after the initial testing). All participants (including the TD children) were tested in a sound-attenuated room in the lab. The order of tasks during testing was consistent for all participants in the WS and VSMA groups; however, the order of tasks was counterbalanced for the VMA controls as they completed behavioural measures as part of a larger neuroimaging study.

### 2.3 Experimental measures

We first describe the measures that were completed by the participants with WS. All typically-developing children performed a subset of these measures, including the same oromotor praxis, nonword repetition and sight word efficiency measures. The VSMA controls also completed the same digit span and oral diadochokinetic tasks as well as a similar tone sequence reproduction measure. Further details on the other tasks completed by the two TD cohorts are described in Krishnan et al. (2013a and under review). Table 1 shows descriptive statistics for all the experimental measures.

Participants with WS completed a brief hearing screening. Pure tone average thresholds ranged between 3 and 57 dB for the left ear and 2 and 42 dB for the right ear. To ensure that our data were not unduly affected by participants' hearing status, all stimuli for our tasks were presented to both ears, or in free-field listening conditions. We established that all our participants had a hearing threshold below 20 dB (considered normal) in at least one ear. We also observed no significant correlations between hearing thresholds in either ear with age, oromotor measures or nonword repetition (*p*>0.15).

#### 2.3.1 Standardized measures

*British Picture Vocabulary Scales* (*BPVS*) II, (Dunn et al., 1997): We used this measure to assess vocabulary levels. A word is spoken, children are shown four line drawings and they have to point to the drawing that best represents the word. This test has been used extensively to match individuals with WS to TD populations (for example, Rhodes et al., 2010).

*Raven*'*s Coloured Progressive Matrices* (*RCPM*), (Raven et al., 1992): We used this measure to assess non-verbal ability in individuals with WS. Here, children are shown a colored matrix with a piece missing. They are given six options to complete the figure, only one of which is correct. Previous assays have shown that this is a well-grounded measure to use for matching individuals with WS to control groups (Van Herwegen et al., 2011).

#### 2.3.2 Measures of phonological proficiency

*Test of Word Reading Efficiency* (*TOWRE*), (Torgesen et al., 1999): The sight word reading efficiency subtest was administered. This is a simple test of fluency, which involves reading a list of progressively more complex words within 45 s. The child's score on this test is the number of words accurately read. In TD school-age children, TOWRE scores are correlated with nonword repetition (Nation and Hulme, 2010).

*Nonword repetition*: The NWR subtest was taken from the Comprehensive Test of Phonological Processing (CTOPP; Wagner et al., 1999). The 18 nonwords from the test (ranging from one to six syllables) were recorded by a native British English speaker and presented to children over headphones (see also Nation and Hulme, 2010; Nation et al., 2010). Children were asked to repeat the word they had just heard. Three practice trials were provided, followed by the 18 test items. Audio recordings of the children's responses were scored. Fractional scores were awarded on the basis of accuracy. An independent researcher scored thirty percent of these recordings. Inter-rater reliability was >0.85.

#### 2.3.3 Measures of praxis and motor ability

*Oromotor praxis* (Alcock et al., 2000; Krishnan et al., 2013a): In this task, participants were seated in front of a computer screen and shown video-recordings of a researcher making non-linguistic oral movements. They were asked to imitate exactly what the researcher did, and their movements were video-recorded.

The oral movements were non-linguistic and occurred in non-linguistic contexts (for example, spreading lips as in smiling, sticking one's tongue out and so on). All movements were produced by a single person. There were two types of stimuli, simultaneous movements (where three articulators had to be moved at the same time and therefore precisely coordinated, for example, round lips, open mouth wide and stick tongue out) and sequential movements (where movements involving three different articulators had to be sequenced, for example, round lips, open mouth and then bring teeth together). In previous studies, these movements have been shown to be more complex than making the same movement repeatedly and they assess aspects of oromotor skill necessary for fluent, coordinated speech. The simultaneous movements we use necessitate the movement of multiple articulators at the same time with precise timing. This allows for the assessment of temporal programming of movements over different articulators. In contrast, the sequential movements allow for the assessment of temporal and spatial programming of articulators.

Simultaneous and sequential movements were crossed with the presence of a five-second silent memory gap between observation and execution of the oral movement. This gap was introduced to unveil potentially subtle differences in reproduction memory (see Diamond, 1985, and Stiles, 2012, for uses of such a gap). Participants were told to imitate the movement only after they heard a pleasant xylophone sound. For the movements without a memory gap, this sound was played immediately, and for the movements with a gap, the xylophone sound was played five seconds after the completion of the video of the oral movement.

Care was taken to ensure that an auditory strategy would be insufficient to succeed, as different visible movements could be associated with the same sound. Three practice trials were given before the set of movements with the memory gap as well as the set of movements without the memory gap. Participants received verbal feedback on their performance on the practice trials. They then imitated ten movements from the set.

The task was scored using the rating system developed by Square-Storer (1989), where each constituent movement in the set could be rated between 0 and 2. A score of 2 was awarded for a completely accurate repetition, 1 for a partially correct repetition or a mis-ordered sequence, and 0 for an incorrect or omitted movement. Each three-part trial therefore received a score between 0 and 6, and over the task, scores could range between 0 and 120. Thirty percent of the videos were randomly chosen to be coded by an independent rater; reliability was ≥0.85 over the four conditions.

*Oral diadochokinesis* (*DDK*): Even though speech fluency is considered a characteristic strength in the profile of individuals with WS, more recent studies suggest that in narrative tasks children with WS have a slower speech rate and produce fewer syllables/minute than mental-age matched TD children (Rossi et al., 2011). It is unclear whether these differences in speed relate to motor ability or difficulties with conceptual planning and lexical retrieval for narrative production. For this reason, we obtained speeded measures of articulation using an oral diadochokinesis task. This task involves repetitive movement of one articulatory movement (for example, “[pa][pa][pa]…”) as quickly as possible, or a performing repetitive motor sequence of three positions as soon as possible.

The child was asked to repeat the syllables [pa], [ta] and [ka] and the trisyllablic sequence [pataka], in each case 12 times, as fast as possible and on a single expiration (Rvachew et al., 2006; Krishnan et al., under review). The task was modeled by the experimenter and followed by a practice trial. Three test trials were recorded. The average length of each syllable or syllable sequence was calculated by dividing the total duration of articulation by the number of syllables (or syllable sequence in the case of [pataka]) in each production. The sequential diadochokinetic rate equals the fastest rate for the syllable sequence [pataka]. The alternating diadochokinetic rate was calculated by averaging the fastest rates for each of the single syllables [pa], [ta] and [ka].

#### 2.3.4 Measures of memory

*Digit Span*: The forward digit span subtest from the Working Memory Test Battery for Children (WMTB-C; Pickering and Gathercole, 2001) was administered. In this task, the participant was required to remember a sequence of digits in the order of their presentation. At each level, the number of digits in a sequence increases by one. A level has six sequences and the participant continues to progress up the levels until >50% of the sequences are incorrect. The participant's score is the final level s/he reaches.

*Tone sequence reproduction*: We assessed non-linguistic sequence reproduction using a task presented on an iPad. This task was akin to SIMON, a popular children's game that is thought to rely on visuospatial working memory (Baniqued et al., 2013). An advantage of using this task was that individuals with WS could respond simply by pressing virtual buttons. The gaming interface comprised a circular arrangement of four buttons. Four tones (262 Hz [C4]; 327.5 Hz [E4]; 393 Hz [G4]; 524 Hz [C5]) were used and each was uniquely paired with a single button at random. These pairings remained fixed for the duration of the game.

At the start of a sequence, a single virtual button was illuminated and the associated tone played. If the participant clicked the correct button, the length of the sequence was expanded by one button+tone. The participant then had to imitate the two-element sequence. The length of the sequence continued to increase until the participant imitated the sequence incorrectly, or until the maximum length of 21 items was reached. The participant's score for the trial was the maximum sequence length they imitated. Participants were given three practice trials after the experimenter explained how to play the game. Participants had to score >3 on each of the trials during practice to proceed; if they failed to meet this criterion, they were given one more practice set. They then completed ten trials, and an average sequence length was calculated for all sequences where sequence length >1.

Since the WS group was tested somewhat later than the TD children, we used an improved version of this memory task with them, but first verifying that it yielded similar results with typical adults. The new version comprised sequences drawn from a probabilistic language consisting of four triplets. In this language, first-order transition probabilities were greater within triplets (*p*=1), compared to between triplets (*p*=0.33). In the older sequences (which were completed by the VSMA controls), there was no triplet structure and first-order probabilities were always 0.25. Pilot data showed that there were no significant differences in completed sequence length for adults completing these two different sets (*p*=0.65).

## 3 Results

Descriptive statistics (means and standard deviation) for all the measures are shown in Table 1.

### 3.1 Oromotor control/imitation skills are weaker in WS relative to VMA and VSMA controls (Fig. 1)

**Fig. 1:**
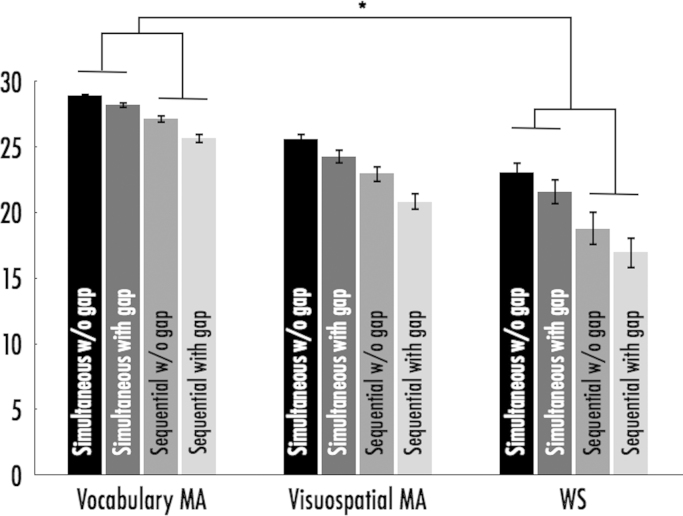
Scores for subscales of the oromotor praxis task; all groups are significantly different from each other. Error bars show ±1 standard error. The interaction between oromotor subscale and group is highlighted in the figure.

When oromotor praxis scores were compared across groups, we found main effects of group F(2,96)=42.463, *p*<0.001, *η*^2^=0.469, oromotor sub-scale (sequential/ simultaneous), *F*(1,96)=98.593, *p*<0.001, *η*^2^=0.507, memory gap (with/without memory gap), *F*(1,96)=39.205, *p*<0.001, *η*^2^=0.290 as well as an interaction between simultaneous/sequential movements and group, *F*(2,96)=4.025, *p*=0.021, *η*^2^=0.077. Bonferroni corrected post-hoc *t*-tests showed that all three groups significantly differed from each other (*p*<0.001) with the VMA controls (*M*=27.49, se=0.54) and VSMA controls (*M*=23.41, se=0.54) more accurate than the WS group (*M*=20.09, se=0.61). The main effect of oromotor sub-scale was driven by higher scores for simultaneous movements (*M*=25.27, se=0.29) relative to sequential movements (*M*=22.06, se=0.43). Further, Bonferroni corrected post-hoc *t*-tests showed that the interaction between oromotor sub-scale and group was driven by the WS group who performed less accurately than VMA controls particularly when given sequential movements rather than simultaneous, *p*=0.017. The main effect of memory gap was driven by greater accuracy when oromotor praxis was assessed without a memory gap (M=24.41, se=0.33) relative to when a memory gap was introduced (*M*=22.92, se=0.36). Fig. 1.

### 3.2 Nonword repetition in WS is poorer than VMA controls, but comparable to VSMA controls (Fig. 2)

**Fig. 2:**
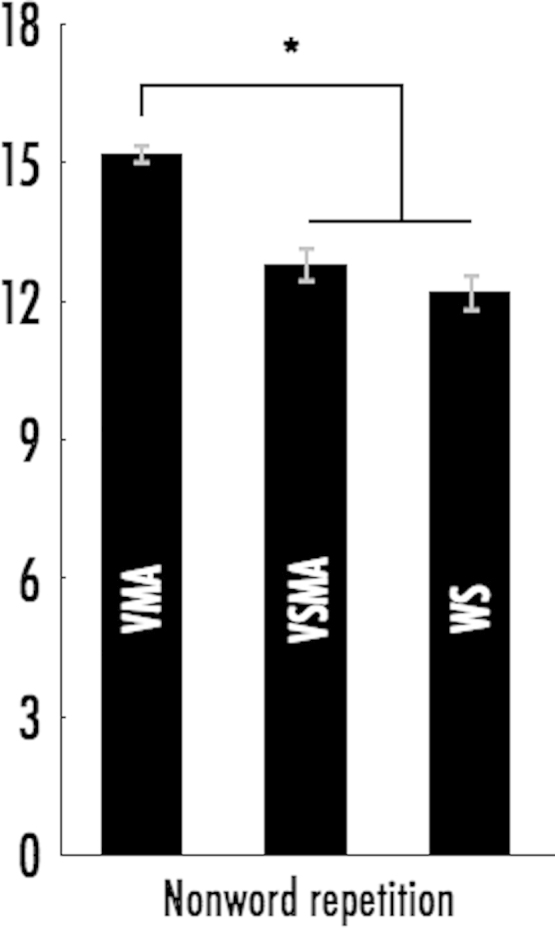
Differences in nonword repetition across VMA controls, VSMA controls and individuals with WS. The WS group is significantly different from the VMA group but not from the VSMA group. Error bars show ±1 standard error.

To ascertain whether individuals with WS had verbal abilities similar to those found in vocabulary-matched TD children, we compared nonword repetition accuracy across our three groups. We found a significant effect of group, *F*(2,101)=20.333, *p*<0.001, *η*^2^=0.291. We followed this up with Bonferroni corrected post-hoc tests, which revealed that the WS group and the VSMA controls were both significantly less accurate than the VMA controls (*p*<0.001), but not significantly different from each other (*p*=0.763) (Fig. 2).

This finding replicates results from Grant et al. (1997) who used a different nonword repetition test (CNRep) and found that performance for children with WS was comparable to a group matched on Raven's ability but that they were significantly poorer than the group matched on vocabulary ability. However, these results do suggest that oromotor abilities of WS should at least be at par with the VSMA controls – which they are not.

### 3.3 Pattern of differences relative to VSMA controls (Fig. 3)

We did not have scores for the VMA controls for the full set of tasks that the participants with WS completed. However, the VSMA controls completed all these tasks. Compared to the VSMA controls, we found that participants with WS had lower scores on the memory measures of digit span, *t*(61)=3.205, *p*=0.002, and tone sequence reproduction, *t*(61)=3.765, *p*<0.001. In contrast, we found no significant differences across these two groups for both alternating (*p*=0.403) and sequential DDK rate (*p*=0.329).

**Fig. 3:**
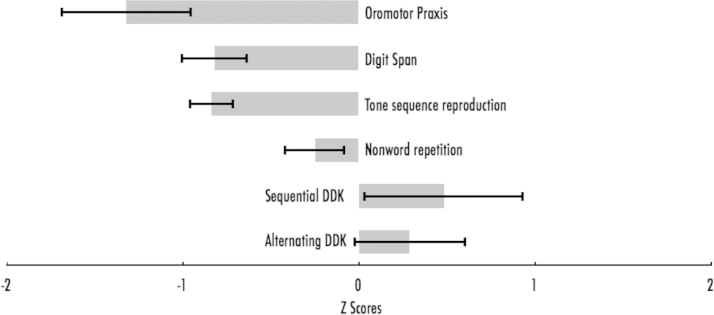
Means and standard errors of *z*-scores. for the WS group relative to VSMA controls. Negative *z*-scores. indicate lower scores than the average of the VSMA group, whereas positive *z*-scores. suggest that scores are higher.

For all the WS participants, *z*-scores for each of these tasks were calculated (relative to the distributions of the VSMA controls) to understand the relative strengths and weaknesses in their cognitive profile. A repeated-measures ANOVA yielded a significant interaction of task, *F*(1,27)=5.335, *p*<0.001. Bonferroni-corrected post-hoc *t*-tests indicated that individuals with WS had higher *z*-scores for nonword repetition relative to their scores on tone sequence reproduction (*p*=0.039) and digit span (*p*=0.048) (Fig. 3).

### 3.4 Summary of findings

We have shown that oromotor praxis scores are lower in the WS group compared to both sets of TD controls. Relative to the younger TD controls, individuals with WS had lower scores on both the digit span and tone sequence reproduction tasks. However, on nonword repetition tasks as well as the diadochokinetic tasks, individuals with WS performed at a level equivalent to that of the VSMA controls.

## 4 Discussion

Oromotor praxis scores were much lower in the WS group compared to both TD controls matched on either vocabulary or visuospatial ability. It is clear that the ability to reproduce novel oral articulatory movements is not in line with the language ability of this group. Moreover, while their performance on the oral DDK and nonword repetition tasks was better than their oromotor praxis, the participants with WS performed only at similar levels to the controls matched on visuospatial ability. This study has revealed for the first time a relative weakness in oromotor praxis for individuals with WS, where they were even less accurate than the VSMA controls. Even in this older group who show relatively good speech production skills (as indexed by oral DDK and nonword repetition), we find that oromotor praxis is not a relative strength. We argue that individual variation in oromotor praxis may play an important role in explaining early delays in language production.

### 4.1 Why would oromotor praxis be a relative weakness in WS?

It is surprising that the WS group performed especially poorly on the oromotor praxis task relative to even the VSMA controls, especially given the fact that their verbal ability was akin to the VMA controls. In fact, in our battery, individuals with WS performed at a level only equivalent to that of non-verbal age-matched controls in two tasks that involved sequencing linguistic units (nonword repetition and oral DDK). Here, we discuss the reasons why oromotor praxis might be a relative weakness in WS.

It is possible that the difficulties faced by participants with WS stem from treating the oromotor praxis task as a spatial sensorimotor transformation task involving changing configurations of the articulators. Indeed, Böhning et al. (2002) have demonstrated that children with WS were impaired when reproducing syllables presented visually but not when they were presented aurally. However, this would not explain why their performance is worse than the VSMA controls. An alternate or additional possibility is that participants with WS have more stable auditory-motor mappings relative to visuomotor mappings, and therefore find it easier to execute these correctly. Such task differences could stem from the support derived from phonological proficiency, which is known to improve over development (Vicari et al., 2004). Enhancements in phonological proficiency and practice articulating linguistic tokens could explain why individuals with WS were better at computing sensorimotor transformations for novel words but not novel non-linguistic oromotor sequences. Further, even though the oral DDK tasks were non-meaningful, participants were reproducing a practised set of syllables from their phonological repertoire.

This explanation would posit that children with WS would also struggle in the initial phases of learning of auditory-motor sequences. Indeed, this may be akin to the difficulties they face when learning visuomotor sequences (Foti et al., 2013; Vicari et al., 2001). Participants with WS may face greater problems with novel and unpracticed visuomotor sequences than TD children because they do not extract and generalise common features from other domains adequately. In this case, they are able to sequence oral movements for speech, but unable to produce similar oral movements to complete the non-linguistic oromotor task. Laing et al. (2002) suggested that when the system is stretched when completing unfamiliar tasks, those with WS tended to show different effects from typically developing children. For example, children with WS did not show concreteness effects in a novel task where printed words had to be associated with their spoken form, but they did show concreteness effects in a spoken nonword repetition task (Laing et al., 2001, 2002). Therefore, it is possible that initial difficulties in oromotor praxis would be present in both auditory-motor and visuomotor tasks. However, these oromotor difficulties could be compensated for in language tasks, perhaps due to repetitive oral practice driven by the intrinsic motivation that children with WS have to speak, or due to improvements in phonological proficiency. However, these difficulties would still be apparent on a novel task that involves computing a slightly different sensorimotor transformation. This account leads to a hypothesis that can be tested in future studies – the children performing poorly on oromotor tasks should also perform poorly tasks that involve generalizing a learnt skill.

An account of the difference between nonword repetition and oromotor praxis must also explain the variation in the performance of the WS participants for the oromotor tasks. Our results indicated that participants with WS closely approximated the performance of the VSMA controls for nonword repetition, and to a greater extent than the two memory tasks (digit span and tone sequence reproduction). However, much greater variability was observed for the motor measures. Rather than an absolute difference, there is likely to be a gradient of difficulty in this group. This suggests that there is more than one factor than contributes to oromotor difficulties. In addition to the problems generalising learnt movements to novel situations, difficulties with motor control, planning and co-ordination in the performance of visuomotor tasks have recently been highlighted in WS (Hocking et al., 2008; Cowie et al., 2011). In a rapid visuomotor aiming task, Elliott et al. (2006) showed that individuals with WS exhibited poor planning processes prior to movement initiation, reflected by their slower peak velocities. Hocking et al. (2011) demonstrated that adults with WS seemed to have particular difficulties in planning and controlling movement under increasing demands of precision. In a later study, Hocking et al. (2013) showed that watching their own movements benefited children with WS in a visuomotor task. The authors suggest that individuals with WS have difficulties updating their limb representations with ongoing proprioceptive feedback. The sensory feedback that is received during these tasks may partially account for differences between visuomotor tasks like oromotor praxis as compared to auditory-motor tasks like nonword repetition and oral DDK. In both auditory-motor tasks, participants received auditory as well as proprioceptive feedback. However, in the oromotor praxis task, participants only receive proprioceptive feedback and they cannot see their own articulation. Such a difference in sensory feedback may be another reason why individuals with WS perform better on linguistic tasks.

Finally, it is unlikely that the difficulties faced by the WS group on the oromotor praxis task relate to short-term memory. Despite the fact that participants with WS performed less accurately than the VSMA controls for the digit span and tone sequence reproduction tasks, the minimum score obtained by the WS participants for both these measures was three units. Our oromotor sequences were short (three elements long). This indicates that it was not specific difficulties with the retention of these three-unit sequences that led to poorer performance. In addition, our group and others have shown that praxis does not pattern with auditory memory (Ayres et al., 1987; Krishnan et al., under review).

### 4.2 Relevance to the neural bases of oromotor praxis

Behavioural differences in oromotor praxis could be related to structural neural differences in WS. Neural regions typically associated with articulatory control and praxis (such as the caudate nucleus and the frontal operculum) are significantly different in WS from TD children. For example, Campbell et al. (2009) note that relative to typical development, children with WS had reduced volumes of the caudate nucleus and putamen as well as reduced gray matter over the left putamen and cerebellum (among other reductions). Meda et al. (2012) observed similar atypicalities in adults with WS, that is, reduced volumes over the basal ganglia circuit including the caudate nucleus, putamen and thalamus. Van Essen et al. (2006) also examined cortical folding and observed that over the frontal operculum, TD children had deeper folds than adults with WS; however, this pattern was reversed over the inferior frontal gyrus. These structures have been linked with speech/motor ability in other populations, most notably, in individuals with developmental verbal dyspraxia (Watkins et al., 2002).

While such brain-behaviour associations might suggest a simple one-to-one relationship, we note that there was considerable inter-individual variability in oromotor praxis within the WS group. For instance, some WS participants did as well as TD children matched on verbal MA. Most studies that describe the WS brain have only compared small groups of individuals with WS to TD groups matched on chronological age. In fact, very few studies have investigated brain-behaviour relationships at the individual level for participants with WS. One possibility is that individuals with WS differ in the extent to which neural circuits associated with speech disorders are affected. However, it is also possible that individuals with WS who have relatively good oromotor praxis/nonword repetition ability use different strategies to compute sensorimotor relationships (for instance, depending more on phonological proficiency) and therefore rely on a different set of neural circuits. Although the current study cannot tease these questions apart, they are clearly relevant to understanding the function of the basal ganglia nuclei and their contribution to motor control in WS.

### 4.3 Variability in oromotor praxis across developmental disorders

Oromotor praxis has been found to be associated with language ability in other developmental disorders. For example, Stark and Blackwell (1997) found that oromotor praxis skills were associated with nonword repetition in children with language impairment. Moreover, fine motor difficulties predict later speech fluency in children with autism (LeBarton and Iverson, 2013), and oromotor praxis is also a problem area for individuals with Down Syndrome (Elliott et al., 1990). The current study has identified underlying oromotor praxis difficulties also in Williams syndrome.

Understanding oromotor praxis in neurodevelopmental disorders could help to delineate differences in developmental trajectories of these different disorders. Both WS and Down syndrome present with considerable and similar early language delays. But the reasons for this may be very different. In Down syndrome, praxic difficulties may occur in combination with more general motor impairments. By contrast, in WS, problems appear to be specific to oromotor praxis, as evidenced by the relatively better performance on nonword repetition and oral DDK tasks. In WS, oral movements for speech might become practised over development (perhaps due to greater imitation of words, social motivation or interest in language in WS), this would lead to the establishment of stable auditory-motor mappings and better speech fluency. Therefore, initial difficulties with oromotor praxis may be tempered by factors that provide resilience during language development, such as phonological proficiency and intrinsic motivation to practice and produce speech. We speculate that oromotor praxis difficulties may account for the *early* language differences in WS (when they look comparable to young children with DS). However, once more stable auditory-motor mappings are formed, children with WS would start to outstrip their peers with DS. However, these early oromotor difficulties would still be apparent when individuals with WS cannot use their established auditory-motor maps.

## 5 Summary and conclusions

In summary, we find that individuals with WS show surprisingly poor imitation and sequencing of complex non-linguistic oral movements as assessed by our oromotor praxis task, relative to TD children matched on either verbal or visuospatial ability. We hypothesize that initial difficulties in planning and coordinating the production of novel oral movements may be one factor contributing to the surprising language delay in early development in Williams syndrome, an issue that the literature has hitherto failed to address.

## Figures and Tables

**Table 1:** Means and standard deviations for all three groups.

	**Williams syndrome**	**VMA controls**	**VSMA controls**
*N*	28	39	35
Chronological Age (years)	16.5 (2.9)	10.1 (1.5)	7.1 (1.0)
Pure Tone Average (Left Ear)	13.3 (10.4)	–	–
Pure Tone Average (Right Ear)	14.0 (9.8)	–	–
BPVS Age	9.8 (2.1)	–	–
Raven's Progressive Matrices Age	7.4 (1.0)	–	–
Reading efficiency (raw scores)	42.7 (22.1)	73 (12.5)	55 (14.7)
Nonword repetition	12.2 (2.3)	15.2 (1.5)	12.8 (2.5)
Oromotor total	80.4 (20.8)	109.9 (4.7)	93.7 (10.1)
Simultaneous without memory gap	23.0 (4.6)	28.9 (1.2)	25.6 (2.7)
Simultaneous with memory gap	21.6 (5.3)	28.2 (1.5)	24.3 (3.4)
Sequential without memory gap	18.8 (7.1)	27.2 (2.1)	23.0 (3.9)
Sequential with memory gap	17.0 (6.4)	25.7 (2.5)	20.8 (4.1)
Alternating DDK rate (syllables/second)	0.22 (0.04)	–	0.21 (0.02)
Sequential DDK rate (trisyllables/second)	0.79 (0.33)	–	0.72 (0.14)
Digit span	4 (0.8)	–	4.7 (0.8)
Tone sequence reproduction	4.4 (0.7)	–	5.3 (1.0)

## References

[bib1] Alcock K. (2006). The development of oral motor control and language. Down Syndr. Res. Pract..

[bib2] Alcock K.J., Krawczyk K. (2010). Individual differences in language development: relationship with motor skill at 21 months. Dev. Sci..

[bib3] Alcock K.J., Passingham R.E., Watkins K.E., Vargha-Khadem F. (2000). Oral dyspraxia in inherited speech impairment and acquired dysphasia. Brain Lang..

[bib4] Ayres A.J., Mailloux Z.K., Wendler C.L. (1987). Developmental dyspraxia: is it a unitary function?. Occup. Ther. J. Res..

[bib5] Baniqued P.L., Lee H., Voss M.W., Basak C., Cosman J.D., DeSouza S., Severson J., Salthouse T.A., Kramer A.F. (2013). Selling points: what cognitive abilities are tapped by casual video games?. Acta Psychol..

[bib9] Böhning M., Campbell R., Karmiloff-Smith A. (2002). Audiovisual speech perception in Williams syndrome. Neuropsychologia.

[bib10] Brock J. (2005). Probed serial recall in Williams syndrome: lexical influences on phonological short-term memory. J. Speech, Lang. Hear. Res..

[bib11] Brock J. (2007). Language abilities in Williams syndrome: a critical review. Dev. Psychopathol..

[bib12] Brookman A., Bishop D.V., McDonald S., McDonald D. (2013). Fine motor deficits in reading disability and language impairment: same or different?. PeerJ.

[bib13] Campbell L.E., Daly E., Toal F., Stevens A., Azuma R., Karmiloff-Smith A. (2009). Brain structural differences associated with the behavioural phenotype in children with Williams syndrome. Brain Res./.

[bib14] Cowie D., Braddick O., Atkinson J. (2011). Visually guided step descent in children with Williams syndrome. Dev. Sci..

[bib15] Diamond A. (1985). Development of the ability to use recall to guide action, as indicated by infants' performance on AB. Child Dev..

[bib16] Donnai D., Karmiloff-Smith A. (2000). Williams syndrome: From genotype through to the cognitive phenotype. Am. J. Med. Genet..

[bib17] Dunn L.M., Dunn L.M., Whetton C., Burley J.N.F.E.R.-Nelson (1997). The British Picture Vocabulary Scale.

[bib18] Elliott D., Weeks D.J., Gray S. (1990). Manual and oral praxis in adults with Down's syndrome. Neuropsychologia.

[bib19] Elliott D., Welsh T.N., Lyons J., Hansen S., Wu M. (2006). The visual regulation of goal-directed reaching movements in adults with Williams syndrome, Down syndrome, and other developmental delays. Motor Control.

[bib20] Ewart A.K., Morris C.A., Atkinson D., Jin W., Sternes K., Spallone P. (1993). Hemizygosity at the elastin locus in a developmental disorder, Williams syndrome. Nat. Genet..

[bib21] Foti F., Menghini D., Mandolesi L., Federico F., Vicari S., Petrosini L. (2013). Learning by observation: insights from Williams syndrome. PLoS ONE.

[bib23] Gernsbacher M.A., Sauer E.A., Geye H.M., Schweigert E.K., Hill Goldsmith H. (2008). Infant and toddler oral‐and manual‐motor skills predict later speech fluency in autism. J. Child Psychol. Psychiatry.

[bib24] Grant J., Paterson S.J., Karmiloff-Smith A., Gathercole S.A., Howlin P., Davies M., Udwin O. (1997). Phonological short-term memory and its relationship to language in Williams syndrome. Cognit. Neuropsychiatry.

[bib01] Havy M., Moukawane S., Nazzi T. (2010). Are 3-to-8-year-old children with Williams syndrome good word-learners?. Neuroreport.

[bib25] Hill E.L. (2001). Non-specific nature of specific language impairment: a review of the literature with regard to concomitant motor impairments. Int. J. Lang. Commun. Disord..

[bib26] Hocking D.R., Bradshaw J.L., Rinehart N.J. (2008). Fronto-parietal and cerebellar contributions to motor dysfunction in Williams syndrome: a review and future directions. Neurosci. Biobehav. Rev..

[bib27] Hocking D.R., Rinehart N.J., McGinley J.L., Moss S.A., Bradshaw J.L. (2011). A kinematic analysis of visually-guided movement in Williams syndrome. J. Neurol. Sci..

[bib28] Hocking D.R., Thomas D., Menant J.C., Porter M.A., Smith S., Lord S.R., Cornish K.M. (2013). The interplay between executive control and motor functioning in Williams syndrome. Dev. Sci..

[bib29] Jarrold C., Baddeley A.D., Hewes A.K. (1998). Verbal and nonverbal abilities in the Williams syndrome phenotype: evidence for diverging developmental trajectories. J. Child Psychol. Psychiatry.

[bib30] Jarrold C., Baddeley A.D., Hewes A.K. (1999). Genetically dissociated components of working memory: evidence from Down's and Williams syndrome. Neuropsychologia.

[bib31] Jarrold C., Brock J. (2004). To match or not to match? Methodological issues in autism-related research. J. Autism Dev. Disord..

[bib32] Kaplan P., Wang P.P., Francke U. (2001). Williams (Williams Beuren) syndrome: a distinct neurobehavioral disorder. J. Child Neurol..

[bib33] Krishnan S., Alcock K.J., Mercure E., Leech R., Barker E., Karmiloff-Smith A., Dick F. (2013). Articulating novel words: children's oromotor skills predict non-word repetition abilities. J. Speech, Lang. Hear. Res..

[bib34] Krishnan S., Leech R., Aydelott J., Dick F. (2013). School-age children's environmental object identification in natural auditory scenes: effects of masking and contextual congruence. Hear. Res..

[bib35] Krishnan, S., Bergstrom, L., Carey, D., Alcock, K.J., Karmiloff-Smith, A., Dick, F. Fractionating Nonword Repetition: The Contribution of Short-term Memory and Oromotor Praxis is different (under review). 10.1371/journal.pone.0178356 PMC5509101 28704379

[bib36] Laing E., Grant J., Thomas M., Ewing S., Karmiloff-Smith A. (2005). Love is… an abstract word: the influence of lexical semantics on verbal short-term memory in Williams syndrome. Cortex.

[bib37] Laing E., Butterworth G., Ansari D., Gsödl M., Longhi E., Panagiotaki G. (2002). Atypical development of language and social communication in toddlers with Williams syndrome. Dev. Sci..

[bib38] Laws G., Bishop D.V. (2004). Pragmatic language impairment and social deficits in Williams syndrome: a comparison with Down's syndrome and specific language impairment. Int. J. Lang. Commun. Disord..

[bib39] LeBarton E.S., Iverson J.M. (2013). Fine motor skill predicts expressive language in infant siblings of children with autism. Dev. Sci..

[bib40] Leech R., Aydelott J., Symons G., Carnevale J., Dick F. (2007). The development of sentence interpretation: effects of perceptual, attentional and semantic interference. Dev.l Sci..

[bib41] Lenhoff H.M., Wang P.P., Greenberg F., Bellugi U. (1997). Williams syndrome and the brain. Sci. Am..

[bib42] Leonard H.C., Hill E.L. (2014). Review: the impact of motor development on typical and atypical social cognition and language: a systematic review. Child and Adolescent Mental Health.

[bib45] Majerus S., Barisnikov K., Vuillemin I., Poncelet M. (2003). An investigation of verbal short-term memory and phonological processing in four children with Williams syndrome. Neurocase.

[bib46] Manly T., Anderson V., Nimmo-Smith I., Turner A., Watson P., Robertson I.H. (2001). The differential assessment of children's attention: the test of everyday attention for children (TEA-Ch), normative sample and ADHD performance. J. Child Psychol. Psychiatry.

[bib47] Masataka N. (2001). Why early linguistic milestones are delayed in children with Williams syndrome: late onset of hand banging as a possible rate-limiting constraint on the emergence of canonical babbling. Dev. Sci..

[bib48] Meda S.A., Pryweller J.R., Thornton-Wells T.A. (2012). Regional brain differences in cortical thickness, surface area and subcortical volume in individuals with Williams syndrome. PLoS ONE.

[bib49] Mervis C.B., Velleman S.L. (2011). Children With Williams syndrome: language, cognitive, and behavioral characteristics and their implications for intervention. Perspect. Lang. Learn. Edu..

[bib51] Morris C.A., Demsey S.A., Leonard C.O., Dilts C., Blackburn B.L. (1988). Natural history of Williams syndrome: physical characteristics. J. Pediatr..

[bib52] Nation K., Hulme C. (2010). Learning to read changes children's phonological skills: evidence from a latent variable longitudinal study of reading and nonword repetition. Dev. Sci..

[bib53] Nation K., Cocksey J., Taylor J.S., Bishop D.V. (2010). A longitudinal investigation of early reading and language skills in children with poor reading comprehension. J. Child Psychol. Psychiatry.

[bib55] Nazzi T., Paterson S.J., Karmiloff-Smith A. (2003). Early word segmentation by infants and toddlers with Williams syndrome. Infancy.

[bib56] Nichols S., Jones W., Roman M.J., Wulfeck B., Delis D.C., Reilly J., Bellugi U. (2004). Mechanisms of verbal memory impairment in four neurodevelopmental disorders. Brain Lang..

[bib57] Paterson S.J., Brown J.H., Gsödl M.K., Johnson M.H., Karmiloff-Smith A. (1999). Cognitive modularity and genetic disorders. Science.

[bib58] Pickering S., Gathercole S.E. (2001). Working memory test battery for children (WMTB-C). Psychol. Corp..

[bib59] Raven J.C., Court J.H., Raven J. (1992). Standard progressive matrices. Manual for Raven's Progressive Matrices and Vocabulary Scales.

[bib60] Reilly J., Klima E.S., Bellugi U. (1990). Once more with feeling: affect and language in atypical populations. Dev. Psychopathol..

[bib61] Rhodes S.M., Riby D.M., Park J., Fraser E., Campbell L.E. (2010). Executive neuropsychological functioning in individuals with Williams syndrome. Neuropsychologia.

[bib62] Ricketts J., Bishop D.V., Nation K. (2007). Vocabulary is important for some, but not all reading skills. Sci. Stud. Read..

[bib02] Robin D.A., Solomon N.P., Moon J.B., Folkins J.W., McNeil M.R. (1997). Nonspeech assessment of the speech production mechanism. Clinical management of sensorimotor speech disorders.

[bib63] Rossi N.F., Sampaio A., Gonçalves ÓF., Giacheti C.M. (2011). Analysis of speech fluency in Williams syndrome. Res. Dev. Disab..

[bib64] Rvachew S., Ohberg A., Savage R. (2006). Young children's responses to maximum performance tasks: preliminary data and recommendations. J. Speech Lang. Pathol. Audiol..

[bib65] Singer-Harris N., Bellugi U., Bates E., Jones W., Rossen M. (1997). Contrasting profiles of language development in children with Williams and Down syndromes. Dev. Neuropsychol..

[bib66] Stark R.E., Blackwell P.B. (1997). Oral volitional movements in children with language impairments. Child Neuropsychol..

[bib67] Stiles J. (2012). The effects of injury to dynamic neural networks in the mature and developing brain. Dev. Psychobiol..

[bib68] Strømme P., Bjørnstad P.G., Ramstad K. (2002). Prevalence estimation of Williams syndrome. J. Child Neurol..

[bib69] Square-Storer P. (1989). Acquired apraxia of speech in aphasic adults.

[bib71] Tsai S.-W., Wu S.-K., Liou Y.-M., Shu S.-G. (2008). Early development in Williams syndrome. Pediatr. Int..

[bib72] Torgesen J.K., Wagner R., Rashotte C. (1999). TOWRE–2 Test of Word Reading Efffciency.

[bib73] Torres E.B., Brincker M., Isenhower R.W., Yanovich P., Stigler K.A., Nurnberger J.I., Yanovich P., Stigler K.A., Nurnberger J.I., Metaxas N.D., José J.V. (2013). Autism: the micro-movement perspective. Front. Integr. Neurosci..

[bib74] Udwin O., Yule W. (1990). Expressive language of children with Williams syndrome. Am. J. Med. Genet..

[bib75] Van Essen D.C., Dierker D., Snyder A.Z., Raichle M.E., Reiss A.L., Korenberg J. (2006). Symmetry of cortical folding abnormalities in Williams syndrome revealed by surface-based analyses. J. Neurosci..

[bib76] Van Herwegen J., Farran E., Annaz D. (2011). Item and error analysis on Raven's coloured progressive matrices in Williams syndrome. Res. Dev. Disab..

[bib77] Vicari S., Brizzolara D., Carlesimo G.A., Pezzini G., Volterra V. (1996). Memory abilities in children with Williams syndrome. Cortex.

[bib78] Vicari S., Bellucci S., Carlesimo G.A. (2001). Procedural learning deficit in children with Williams syndrome. Neuropsychologia.

[bib79] Vicari S., Caselli M.C., Gagliardi C., Tonucci F., Volterra V. (2002). Language acquisition in special populations: a comparison between Down and Williams syndromes. Neuropsychologia.

[bib80] Vicari S., Bates E., Caselli M.C., Pasqualetti P., Gagliardi C., Tonucci F., Volterra V. (2004). Neuropsychological profile of Italians with Williams syndrome: an example of a dissociation between language and cognition?. J. Int. Neuropsychol.Soc..

[bib81] Wagner R.K., Torgesen J.K., Rashotte C.A. (1999). Comprehensive Test of Phonological Processing: CTOPP.

[bib82] Wang P.P., Bellugi U. (1994). Evidence from two genetic syndromes for a dissociation between verbal and visual-spatial short-term memory. J. Clin. Exp. Neuropsychol..

[bib83] Watkins K.E., Friston K., Vargha-Khadem F., Ashburner J., Passingham R.E., Connelly A. (2002). MRI analysis of an inherited speech and language disorder: structural brain abnormalities. Brain.

[bib84] Wechsler D. (1996). Wechsler Objective Numerical Dimensions.

[bib03] Ziegler W. (2002). Task-Related Factors in Oral Motor Control: Speech and Oral Diadochokinesis in Dysarthria and Apraxia of Speech. Brain and Language.

